# Epigenetic silencing of *MEIS2* in prostate cancer recurrence

**DOI:** 10.1186/s13148-019-0742-x

**Published:** 2019-10-22

**Authors:** Maibritt Nørgaard, Christa Haldrup, Marianne Trier Bjerre, Søren Høyer, Benedicte Ulhøi, Michael Borre, Karina D. Sørensen

**Affiliations:** 1 Department of Molecular Medicine, Aarhus University Hospital, Aarhus, Denmark; 2 Department of Clinical Medicine, Aarhus University, Aarhus, Denmark; 3 Department of Urology, Aarhus University Hospital, Aarhus, Denmark; 4 Department of Histopathology, Aarhus University Hospital, Aarhus, Denmark

**Keywords:** Prostate cancer, DNA methylation, RNA expression, MEIS2, Epigenetic silencing, Biomarker, Prognosis

## Abstract

**Background:**

Current diagnostic and prognostic tools for prostate cancer (PC) are suboptimal, resulting in overdiagnosis and overtreatment of clinically insignificant tumors. Thus, to improve the management of PC, novel biomarkers are urgently needed.

**Results:**

In this study, we integrated genome-wide methylome (Illumina 450K DNA methylation array (450K)) and RNA sequencing (RNAseq) data performed in a discovery set of 27 PC and 15 adjacent normal (AN) prostate tissue samples to identify candidate driver genes involved in PC development and/or progression. We found significant enrichment for homeobox genes among the most aberrantly methylated and transcriptionally dysregulated genes in PC. Specifically, homeobox gene *MEIS2* (Myeloid Ecotropic viral Insertion Site 2) was significantly hypermethylated (*p* < 0.0001, Mann-Whitney test) and transcriptionally downregulated (*p* < 0.0001, Mann-Whitney test) in PC compared to non-malignant prostate tissue in our discovery sample set, which was also confirmed in an independent validation set including > 500 PC and AN tissue samples in total (TCGA cohort analyzed by 450K and RNAseq). Furthermore, in three independent radical prostatectomy (RP) cohorts (*n* > 700 patients in total), low MEIS2 transcriptional expression was significantly associated with poor biochemical recurrence (BCR) free survival (*p* = 0.0084, 0.0001, and 0.0191, respectively; log-rank test). Next, we analyzed another RP cohort consisting of > 200 PC, AN, and benign prostatic hyperplasia (BPH) samples by quantitative methylation-specific PCR (qMSP) and found that *MEIS2* was significantly hypermethylated (*p* < 0.0001, Mann-Whitney test) in PC compared to non-malignant prostate tissue samples (AN and BPH) with an AUC > 0.84. Moreover, in this cohort, aberrant *MEIS2* hypermethylation was significantly associated with post-operative BCR (*p* = 0.0068, log-rank test), which was subsequently confirmed (*p* = 0.0067; log-rank test) in the independent TCGA validation cohort (497 RP patients; 450K data).

**Conclusions:**

To the best of our knowledge, this is the first study to investigate, demonstrate, and independently validate a prognostic biomarker potential for MEIS2 at the transcriptional expression level and at the DNA methylation level in PC.

## Background

Prostate cancer (PC) is the most common non-cutaneous cancer among men in the Western world [1]. Early detection of PC is critical, as localized PC is curable by radiation therapy or radical prostatectomy (RP), whereas metastatic disease is lethal with only palliative treatments available. Furthermore, accurate risk stratification at diagnosis is important for treatment decisions, as some PCs remain latent throughout the lifetime of the patients while others may progress to aggressive metastatic disease. However, the currently available diagnostic and prognostic tools for PC are suboptimal, and novel biomarkers are urgently needed to reduce overdiagnosis and overtreatment of clinically insignificant PCs [2].

A molecular hallmark for PC is aberrant DNA methylation of CpG island-containing gene promoters [3]. Promoter hypermethylation is associated with transcriptional repression of, e.g., tumor suppressor genes, whereas promoter hypomethylation is linked with activation of, e.g., oncogenes [4]. Thus, aberrant DNA methylation may play an important role in driving PC oncogenesis and/or progression when affecting the corresponding transcript expression. Several epigenetic candidate diagnostic markers for PC have been discovered through comparison of DNA methylation alterations in PC and non-malignant prostate tissue samples [5–10, 12]. Some of these methylation marker candidates have also shown prognostic potential for prediction of time to biochemical recurrence (BCR) [5, 6, 9, 11, 12]. In the present study, we investigated the diagnostic and prognostic biomarker potential of *MEIS2* (Myeloid Ecotropic viral Insertion Site 2) in relation to PC. *MEIS2* was selected as candidate gene because we found it to be significantly hypermethylated and downregulated in PC compared to non-malignant prostate tissue samples analyzed by genome-wide methylome and transcriptome profiling (see below).

*MEIS2* is a homeobox gene and part of the TALE (three amino acid loop extensions) family of proteins. TALE proteins are a subtype of homeobox proteins that bind to HOX proteins and specify their transcriptional activity [13]. MEIS2 can bind to the domain of HOXB13 that includes the G84E mutation, which in turn has been associated with elevated PC risk and aggressiveness, although it remains unclear how this mutation may affect the HOXB13-MEIS2 interaction and possibly promote the initiation and/or progression of PC [13–15]. Moreover, previous PC studies have shown that MEIS2 transcriptional and protein expression levels decrease gradually from non-malignant prostate to primary PC and to metastatic PC tissue samples, suggesting that MEIS2 plays a tumor suppressive role and may be involved in PC development and/or progression [16, 17]. Similarly, downregulated MEIS2 protein expression has been associated with poor overall survival in a small cohort of 83 PC patients [16]. Another study has suggested *MEIS2* as an important component of a signaling circuit with IκBα/NF-κB (p65), miR-196b-3p, and PPP3CC (protein phosphatase 3 catalytic subunit gamma) that is involved in progression to castration resistant PC (CRPC) [18]. Together, these previous studies indicate a prognostic potential of *MEIS2* in PC, but until now, only protein expression of *MEIS2* has been investigated for its prognostic potential in two cohorts [16, 17].

In the present study, in an effort to identify novel candidate biomarkers for PC, we combined DNA methylation (Illumina 450K DNA methylation array (450K)) and matched RNA expression (RNA sequencing (RNAseq)) data from a set of PC and adjacent normal (AN) tissue samples from 29 patients treated by RP. Among the most differentially methylated and differentially expressed genes, we found a significant overrepresentation of homeobox and homeodomain-containing genes, including *MEIS2* that was hypermethylated and downregulated in PC, indicating epigenetic silencing. Furthermore, low transcriptional expression and DNA hypermethylation of *MEIS2* was significantly associated with BCR after RP in multiple large independent RP cohorts including > 700 PC patients in total. This is the first study to investigate, demonstrate, and independently validate a prognostic biomarker potential for MEIS2 transcriptional expression and DNA methylation in PC.

## Materials and methods

### Patient samples used for RNAseq and 450K methylation profiling (discovery)

For the discovery set, radical prostatectomy (RP) tissue specimens from 29 PC patients (Additional file 1) treated at the Department of Urology between May 2003 and October 2012 (Aarhus University Hospital, Denmark) were obtained immediately after surgery and stored at − 80 °C (fresh frozen in TissueTek). Hematoxylin and eosin (HE) stained prostate tissue sections were evaluated by a trained pathologist and adjacent normal (AN) and PC (tumor) areas were marked for laser capture microdissection (LCM, Veritas^TM^ 704 (Arcturus)). For each sample, after LCM of the top 15–25 6-μm sections, total RNA (> 200 bp) was extracted using the RNeasy micro Kit (Qiagen) according to the manufacturer’s instructions. RNA concentration and RNA quality was assessed using the Agilent RNA 6000 Pico Chip on an Agilent 2100 Bioanalyzer (RIN ≥ 6). Similarly, after LCM of the next 15–25 6-μm sections, genomic DNA was extracted using the Puregene system (Qiagen) according to the manual provided by the manufacturer. DNA concentrations were assessed using the Quant-iT PicoGreen dsDNA Assay Kit (Life Technologies).

### RNA sequencing (RNAseq) and Illumina 450K DNA methylation array (450K)

For RNAseq, directional indexed libraries were generated from 10–500 ng total RNA (> 200 bp) from 42 samples (29 PC samples and 13 AN samples) using the Scriptseq™ Complete Gold Kit Version II (Illumina). RNAseq libraries were sequenced on the Illumina HiSeq2000 (15–25 million reads/sample, 2 × 150 bp), and reads were mapped to the human genome (hg19) using the Tuxedo Suite [19]. Counts were calculated using HTSeq [20]. RNAseq data was analyzed in R version 3.1.2 with the *EdgeR* package version 3.8.5 [21] using counts as input. A total of seven RNA samples (6 PC and 1 AN) had a poor library profile and were removed prior to the final data analysis. Moreover, one tumor sample had a low PC cell content and was also removed prior to analysis.

Genomic DNA from 43 samples (28 tumor samples and 15 AN samples) was bisulfite converted using the EpiTect Bisulfite Kit (Qiagen) and applied to the Illumina 450K DNA methylation array (450K) by service provider Aros Applied Biotechnology A/S (Aarhus, Denmark). Raw 450K DNA methylation array data was analyzed in R version 3.1.2, using the *Chip Analysis Methylation Pipeline* (ChAMP) package version 1.4.0 [22]. Throughout the analyses, the argument *filterXY* was set to FALSE to ensure that probes from X and Y chromosomes were not removed. DNA methylation was reported as *β*-values (range 0-1; 0, unmethylated; 1, completely methylated). One tumor sample (also analyzed by RNAseq) had a low PC cell content and was removed prior to data analysis.

For candidate biomarker discovery, 450K DNA methylation and RNAseq datasets were merged by ENSEMBL gene name annotations, and a combined *p* value was calculated using Fisher’s method [23]. Among the genes with a significant Benjamini-Hochberg (BH)-adjusted Fishers *p* value, The *Functional Annotation Clustering* tool from the *Database for Annotation, Visualization and Integrated Discovery* (DAVID) [24, 25] was used to identify enriched annotation terms (GO, KEGG, etc.). Furthermore, Spearman’s correlations between DNA methylation and RNA expression levels were calculated for each CpG site. DAVID analyses were also performed on the top 3000 differentially methylated genes (BH adj. *p* < 0.05) and for the top 2314 significantly differentially expressed genes (BH adj. *p* < 0.05), respectively.

### Patient samples used for quantitative methylation specific PCR analyses (validation)

For validation, we used quantitative methylation-specific PCR (qMSP) to analyze a RP cohort of 264 patients treated for histologically verified clinically localized PC at Department of Urology, Aarhus University Hospital, Denmark, from 1999 to 2013. In all cases, a trained pathologist evaluated formalin-fixed paraffin-embedded (FFPE) archived prostatectomy specimens, and 1.5-mm punch biopsies were taken from representative regions with cancer (*n* = 254) or AN tissue (*n* = 37) and used for extraction of genomic DNA (see below) [5, 6, 8, 12]. As non-PC controls, FFPE transurethral resection of the prostate (TURP) tissue samples from benign prostatic hyperplasia (BPH, *n* = 9) patients were also included. A total of 82 samples were excluded due to insufficient DNA quality (see below). The final analysis included 195 PC, 17 AN, and 6 BPH samples (Table 1).

**Table 1:** Characteristics of the RP patients used for qMSP

Variable	qMSP cohort (*n* = 195 PC patients)	
Age at RP, median (range)	64 (49–77)	
Pre-operative PSA (ng/mL), median (range)	13 (2.1–284.0)	
Pathological Gleason Score		
< 7, *n* (%)	64 (32.8)	
= 7, *n* (%)	94 (48.2)	
3 + 4, *n* (%)	86 (91.5)	
4 + 3, *n* (%)	8 (8.5)	
> 7 *n* (%)	35 (18.0)	
Unknown, *n* (%)	2 (1.0)	
Pathological T-stage		
≤ pT2c, *n* (%)	126 (64.6)	
≥ pT3a, *n* (%)	67 (34.4)	
Unknown, *n* (%)	2 (1.0)	
Pathological N-stage		
pN0, *n* (%)	167 (85.6)	
pN1, *n* (%)	15 (7.7)	
pNX/unknown, *n* (%)	13 (6.7)	
Surgical margin status		
Negative margin, *n* (%)	135 (69.2)	
Positive margin, *n* (%)	56 (28.7)	
Unknown, *n* (%)	4 (2.1)	
CAPRA-S score		
0–2	54 (27.7)	
3–5	77 (39.5)	
6–10	50 (25.6)	
Unknown	14 (7.2)	
Follow-up (months), median (range)	131 (12–219)	
No PSA recurrence, *n* (%)	88 (45.1)	
PSA recurrence, *n* (%)	104 (53.3)	
Unknown, *n* (%)	3 (1.6)	
Variable	AN (*n* = 17)	BPH (*n* = 6)
Age at RP, median (range)	65 (56–73)	67 (56–73)

### Quantitative methylation specific PCR (qMSP)

DNA was extracted from FFPE punch biopsies of RP specimens (PC and AN) and TURP specimens (BPH) with the gDNA Eliminator columns from the RNeasy plus micro kit (Qiagen) and bisulfite converted using the EZ-96 DNA Methylation-Gold Kit™ (Zymo Research), as previously described in detail [5, 6, 11]. For qMSP assay design, Primer3 [26, 27] and Beacon DesignerTM (Premier Biosoft) were used. Primer and probe sequences are given in Additional file 2. *MEIS2* assay 1 targeted an intronic region of the *MEIS2* gene (intron between exon 4 and 5, Fig. 3a), which overlapped three significant differentially methylated probes on the 450K array (cg06933370, cg23677243, and cg26708220, Fig. 3a). *MEIS2* assay 2 targeted the promoter region of *MEIS2* and overlapped one probe from the 450K array (cg25381383) (Fig. 3a). All qMSP reactions were run in triplicates (10 μL) with 5 ng bisulfite-converted DNA, 6 pmol of each primer, 2 pmol probe, and 5 μL Taqman universal Mastermix no UNG (Applied Biosystems). As controls, standard curves on serially diluted methylated DNA, bisulfite-converted CpGenome Universal Methylated DNA (Millipore), and two negative controls (H_2_O and whole-genome amplified (WGA) DNA) were included on each plate. For quality/quantity control, aluC4 and *MYOD1* assays were used [5]. AluC4 was used for normalization. Reactions were run in 384-well plates on the ViiA7 Real-Time PCR system (Applied Biosystems): 2 min at 50 °C, 10 min at 95 °C, and 40 cycles of 15 sec at 95 °C and 1 min at 56 °C. Quantities for *MEIS2* assay 1 and 2, *MYOD1*, and aluC4 were estimated from the standard curves using QuantStudio™ Real-Time PCR Software (Applied Biosystems). Outliers (more than 2 ct values lower/higher than the ct value of the other replicates) and samples with *MYOD1* ct > 38.0 in ≥ 2 of 3 replicate reactions were removed. For *MEIS2* assay 2, replicates exceeding ct 38 were set to 0 (WGA cutoff). Samples were considered negative for methylation, if ≥ 2 methylation-specific reactions did not amplify.

### Public cohorts (external validation)

#### Long cohort

RNAseq and clinical data for formalin-fixed paraffin-embedded (FFPE) PC tissue from 106 RP patients from Long et al. [28] was downloaded from GEO (GSE54460).

#### TCGA cohort

From The Cancer Genome Atlas (TCGA, http://cancergenome.nih.gov/), we downloaded RNA sequencing (RNAseq), 450K DNA methylation, and clinical data for 497 RP (PC) and 52 matched AN fresh frozen tissue samples [29]. 450K data was peak corrected [30] and RNAseq data was mapped to hg19 and processed as previously described [8]. DNA methylation was reported as *β*-values and RNAseq gene expression as counts per million (CPM). For external validation of our qMSP data, the average methylation level of the 450K probes cg06933370, cg23677243, and cg26708220 was calculated for each patient to mimic qMSP assay 1. qMSP assay 2 covered one probe from the 450K array, cg25381383, which was used for external validation of assay 2.

#### Taylor cohort

Normalized microarray (Affymetrix Human Exon 1.0 ST array) RNA expression and clinical data for fresh frozen PC tissue from 126 RP samples were downloaded from GEO (GSE21034) [31].

#### Statistical analysis

*A*ll statistical analyses were conducted in STATA version 13.1. To investigate the diagnostic and prognostic potential of *MEIS2* DNA methylation and transcriptional expression, we used Mann-Whitney tests, ROC curve analyses, uni- and multivariate Cox regression analyses, Kaplan-Meier, and log-rank tests. In time-to-event analysis, PSA recurrence (cutoff ≥ 0.2 ng/mL) was used as endpoint. In all datasets, dichotomization of patients into high- and low-risk groups based on either MEIS2 expression or *MEIS2* methylation levels was made by ROC curve analyses of BCR status at 36 months of follow-up. For the qMSP assays, the cutoffs for dichotomization were 0.109/0.162, respectively. For the *MEIS2* methylation model, patients were included in the low methylation group if both qMSP assay 1 and qMSP assay 2 showed low methylation.

## Results

### Methylome and transcriptome profiling of PC tissue for biomarker discovery

**Fig. 1:**
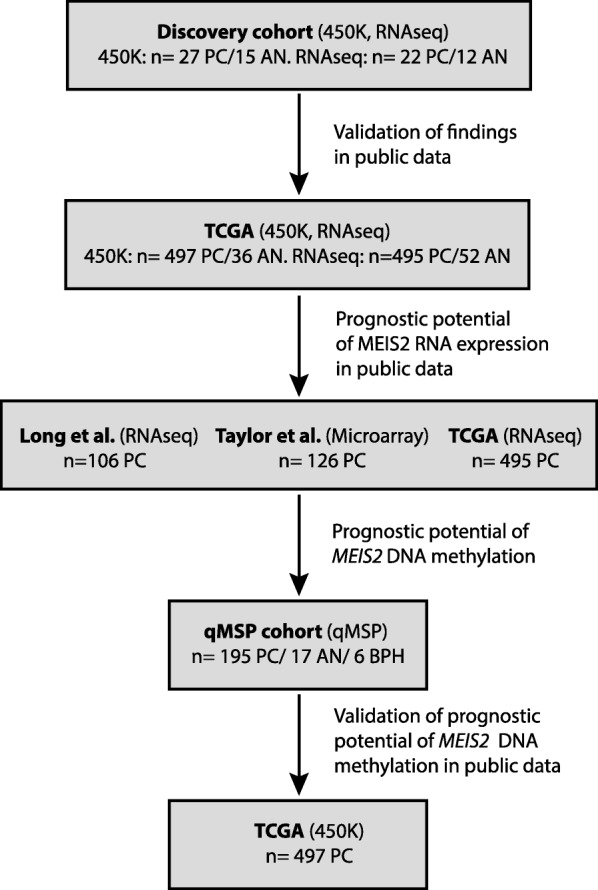
Flowchart of the datasets used. For each step, the datatype and number of samples used for the analyses are indicated. PC, prostate cancer; AN, adjacent normal; BPH, benign prostatic hyperplasia; qMSP, quantitative methylation specific PCR; 450K, Illumina 450K DNA methylation array

To identify novel epigenetically and transcriptionally deregulated biomarker candidates, we performed RNAseq and 450K DNA methylation analysis on microdissected PC and AN tissue samples from RP specimens. After quality control (see “Materials and methods” section), the final 450K dataset consisted of 27 PC and 15 AN samples and the final RNAseq dataset of 22 PC and 12 AN samples (Fig. 1). A total of 22 PC and 12 AN samples were analyzed by both methods.

The 450K analysis identified 119,519 CpG sites with a significant Benjamini-Hochberg (BH)-adjusted *p* value when comparing methylation in PC and AN samples (BH adj. *p* < 0.05, *t* statistics in ChAMP-package), corresponding to differential methylation at approx. 25% of all CpG sites analyzed. In a multi-dimensional scaling (MDS) plot of the 1000 most variable CpG sites (Additional file 3), PC and AN samples clustered separately, demonstrating that DNA methylation levels are substantially altered in PC tissue samples, consistent with previous reports [3, 5]. Moreover, by RNAseq, we found that transcriptional expression of 2314 genes was significantly deregulated in PC vs. AN samples after adjusting for multiple testing (BH adj. *p* < 0.05, exactTest in EdgeR package (negative binomial test)). The corresponding MDS plot of the 150 most variable transcripts clearly separated PC and AN samples (Additional file 3). As a technical validation of RNAseq results, RT-qPCR of MEIS2 on 7 prostate cell lines (2 benign and 5 malignant) and primary prostate epithelial cells was performed and showed that RT-qPCR results were highly comparable to RNAseq results (data not shown; Spearman’s rho 1.00, *p* < 0.001).

**Fig. 2:**
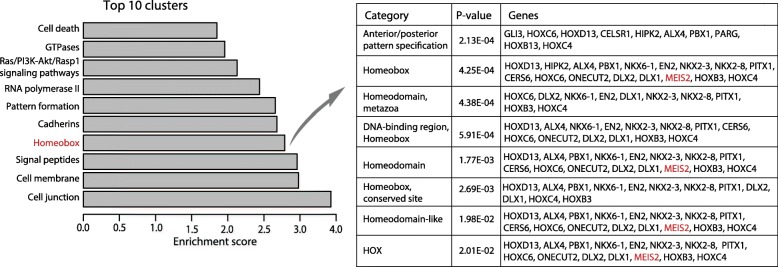
DAVID functional annotation analysis. Top 10 enriched clusters identified by DAVID functional annotation analysis on 1125 genes with a significant BH-adjusted combined Fisher’s *p* value. Left*:* Barplot showing enrichment score of top 10 clusters. A general term describing the genes/categories within each cluster is given. Right: Detailed list for the Homeobox cluster with *p* values and genes for each category

Next, to identify epigenetically deregulated gene expression, 450K and RNAseq datasets were merged by gene name and combined *p* values (Fisher’s *p* values) were calculated from the *p* values obtained by comparing PC and AN samples for each data type. A total of 1125 genes had a combined BH-adjusted *p* value < 0.05, indicating significant deregulation in DNA methylation and/or RNA expression levels in PC tissue samples. Functional annotation analysis using DAVID [24, 25] revealed significant enrichment for homeobox and homeodomain-containing genes (enrichment score of homeobox cluster, 2.79; the 4th most enriched cluster; Fig. 2) in our list of significantly epigenetically deregulated genes in PC. The homeobox cluster included genes such as *HOXC6* and *DLX1* which are included in the SelectMDx® urine-based test for detection of high-risk PC [32], as well as *EN2*, *GLI3*, and *MEIS2* previously investigated for their biomarker potential and/or function in PC [18, 33, 34]. Functional annotation analyses on the top 3000 differentially methylated and 2314 differentially expressed genes, respectively, overall yielded similar results compared to the 1125 epigenetically deregulated genes (Additional file 4). Moreover, the homeobox clusters were the 6th (methylation) and 51st (expression) most enriched clusters, respectively, suggesting that transcriptional expression is not altered in all differentially methylated homeobox genes.

### DNA methylation and RNA expression of MEIS2

**Fig. 3:**
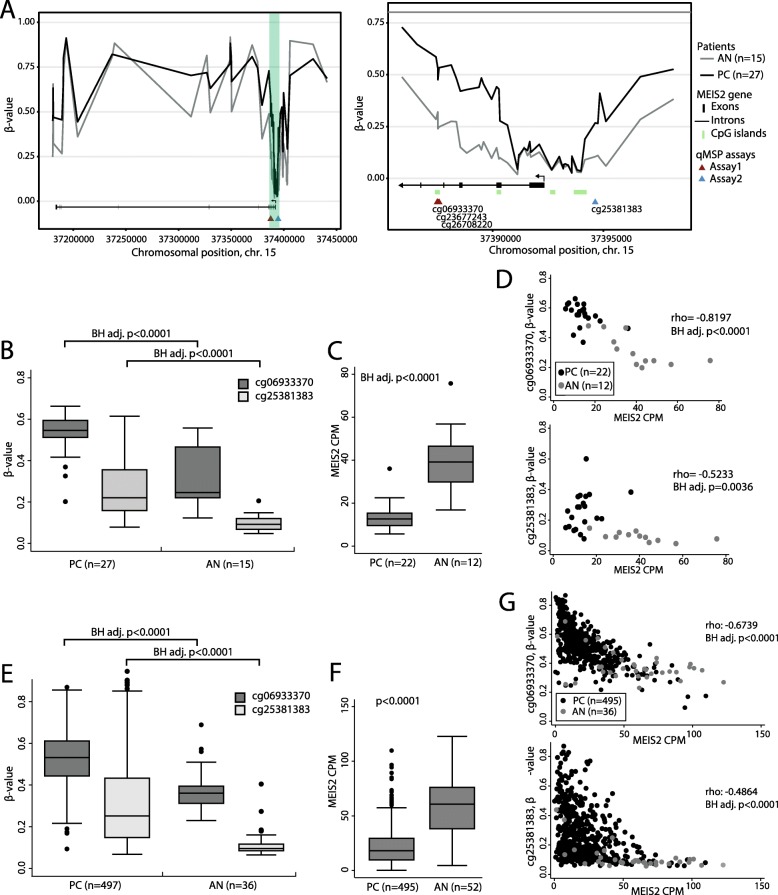
DNA methylation and transcriptional expression of *MEIS2* in the discovery and TCGA cohorts. **a Left**: Mean DNA methylation across the *MEIS2* gene in the discovery cohort (27 PC (black)/15 AN (gray) samples. 450K). **Right***:* Zoom in on promoter region of ***MEIS2*** (green-box in left plot). **b** DNA methylation of cg06933370 and cg25381383 in the discovery cohort (27 PC/15 AN samples from 450K). **c** MEIS2 RNA expression in the discovery cohort (22 PC/12 AN samples. RNAseq). **d** Spearman's correlation between  DNA methylation of cg06933370 (covered by qMSP assay 1, see Fig. **a**) or cg25381383 (covered by qMSP assay 2, see a) and MEIS2 RNA expression in the discovery cohort (22 PC (black)/12 AN samples (gray)). **e** DNA methylation of cg06933370 and cg25381383 in the TCGA cohort (497 PC/36 AN samples. 450K). **f** MEIS2 expression in the TCGA cohort (495 PC/52 AN samples; RNAseq). g Correlation between DNA methylation of cg06933370 or cg25381383 and MEIS2 RNA expression in TCGA data (495 PC (black)/36 AN (gray)). PC, prostate cancer; AN, adjacent normal; CPM, counts per million; Chr., chromosome; BH adj., Benjamini-Hochberg adjusted; P, *p* value

Next, to assess correlations between DNA methylation and transcriptional expression, Spearman’s rho coefficients were calculated for each individual CpG site and its corresponding gene (using the 34 PC and AN samples analyzed by both methods). This analysis revealed significant differential methylation as well as downregulation of *MEIS2*, a homeobox gene previously investigated for its tumor suppressive role in PC initiation and progression [16–18]. More specifically, in the 450K data, *MEIS2* was significantly hypermethylated in PC samples both in the promoter region and from exon 2–5 (cg06933370, BH adj. *p* < 0.0001; cg25181383, BH adj. *p* < 0.0001; Mann-Whitney test; Fig. 3a, b) and the transcriptional expression of MEIS2 was significantly downregulated in PC compared to AN samples in the RNAseq dataset (BH adj. *p* < 0.0001, Mann-Whitney test; Fig. 3c), consistent with epigenetic silencing through promoter hypermethylation. Moreover, we found a significant inverse correlation between MEIS2 transcriptional expression and DNA methylation both in the promoter and intronic region (cg25381383 (promoter region)/cg06933370 (intronic region), rho = − 0.5233/− 0.8197, BH adj. *p* = 0.0036/*p* < 0.0001; Spearman correlation; Fig. 3d).

This finding was subsequently confirmed in a large independent RP cohort from TCGA including 497 PC and 52 AN samples with both 450K and RNAseq data available [29]. *MEIS2* was significantly hypermethylated (cg06933370 and cg25381383, BH adj. *p* < 0.0001, Mann-Whitney test; Fig. 3e) and downregulated (*p* < 0.0001, Mann-Whitney test; Fig. 3f) in PC samples compared to AN samples in the TCGA cohorts. Likewise, DNA methylation of cg06933370 and cg25381383 was significantly inversely correlated with MEIS2 transcriptional expression also in the large TCGA cohort (rho = − 0.6739 and − 0.4864, BH adj. *p* < 0.0001; Spearman correlation; Fig. 3g).

Together, these results strongly indicate that aberrant DNA hypermethylation is associated with epigenetic silencing of MEIS2 transcriptional expression in PC.

### Prognostic potential of MEIS2 RNA expression

**Fig. 4:**
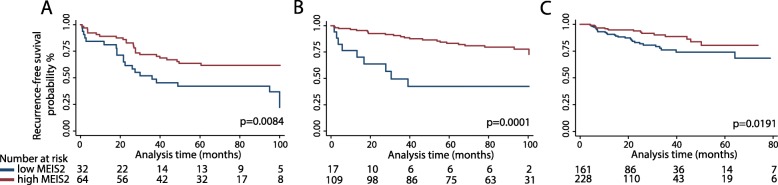
Prognostic potential of MEIS2 RNA expression in three RP cohorts. Dichotomization of patients into low and high MEIS2 RNA expression groups was based on ROC curves of BCR status at 36 months follow-up (not shown). Kaplan-Meier BCR-free survival estimate of low/high MEIS2 RNA expression in three RP cohorts: **a** Long et al. (RNAseq), **b** Taylor et al. (microarray), and **c** TCGA (RNAseq). *p* values were calculated using log-rank tests

Next, to test the possible prognostic potential of MEIS2 at the transcriptional level, we used data from three publicly available PC patient cohorts: RNAseq data from Long et al. (*n* = 106 RP patients) [28], microarray expression data from Taylor et al. (*n* = 126 RP patients) [35], and RNAseq data from TCGA (*n* = 495 RP patients) [29]. Low MEIS2 expression was generally associated with unfavorable clinicopathological parameters (high pathological Gleason score, advanced pathological T stage, and/or positive surgical margins), although this was only statistically significant for Gleason score in the Taylor and TCGA cohort, and for pathological T-stage in the TCGA cohort (Additional file 5). Furthermore, in all three RP cohorts, low MEIS2 RNA expression was significantly associated with poor BCR-free survival in Kaplan-Meier (log-rank test, *p* = 0.0084, 0.0001, and 0.0191, respectively; Fig. 4a–c) and univariate cox regression analyses (*p* = 0.010, 0.000, and 0.022), respectively (Additional files 6, 7 and 8). After adjustment for routine clinicopathological parameters, MEIS2 expression remains a significant predictor of BCR only in the Long cohort (*p* = 0.005, HR = 0.39 (0.20–0.76); Additional file 6). Similar results were obtained when analyzing MEIS2 transcriptional expression as a continuous variable (Additional files 6, 7 and 8).

Together, these results indicate that low MEIS2 transcriptional expression is associated with more aggressive PC, thereby expanding on a previous report that linked low MEIS2 protein levels with poor overall survival in PC [16].

### Prognostic potential of *MEIS2* DNA methylation

**Fig. 5:**
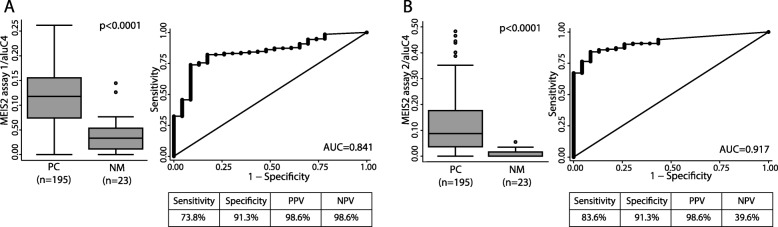
Diagnostic potential of *MEIS2* methylation (assay 1 and 2) in PC vs. AN and BPH samples in the qMSP cohort. Left: Box plots of *MEIS2* methylation levels in PC and NM samples (AN and BPH). Right: ROC curves of data presented in box plots. **a** MEIS2 qMSP assay 1 (including cg06933370, cg23677243, and cg26708220, see Fig. 3a). **b** MEIS2 qMSP assay 2 (including cg25381383, see Fig. 3a). P, ***p*** value; AUC, area under the curve; NM, non-malignant; PPV, positive predictive value; NPV, negative predictive value

**Fig. 6:**
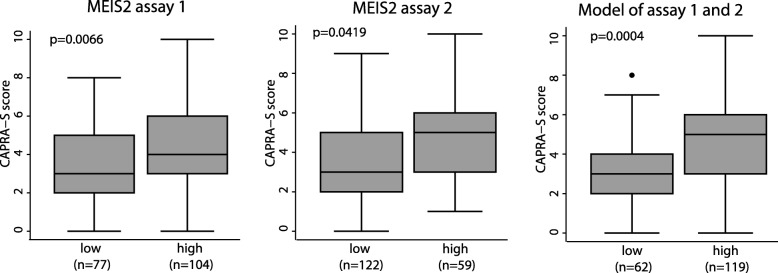
Association between low and high methylation of *MEIS2* in the qMSP cohort and CAPRA-S score. Patients were dichotomized based on BCR status at 36 months. CAPRA-S score was not available for all patients (unknown, *n* = 14). *p* values were calculated with Mann-Whitney test. P, *p* value

Next, to examine the prognostic potential of *MEIS2* DNA methylation for prediction of post-operative BCR, we designed two qMSP assays each covering a significantly hypermethylated region of the *MEIS2* gene (intragenic and promoter, respectively) identified above by 450K analysis (Fig. 3a, b). Thus, qMSP assay 1 covered three probes from the 450K array within the *MEIS2* gene (cg06933370, cg23677243, and cg26708220) and assay 2 covered one probe from the 450K array in the promoter region of *MEIS2* (cg25381383). qMSP analyses were performed on an independent set of 195 PC, 17 AN, and 6 BPH samples (Table 1). We combined the AN and BPH samples into one non-malignant sample group, as there were no significant difference in *MEIS2* methylation between these sample types. For both assays, *MEIS2* was significantly hypermethylated in PC tissue samples (*p* < 0.0001, Mann-Whitney test; Fig. 5a, b left) and showed promising diagnostic potential with AUCs of 0.841 and 0.917, respectively (Fig. 5a, b right). For both assays, high *MEIS2* DNA methylation was generally associated with adverse clinicopathological factors (high pathological Gleason score, advanced pathological T stage, positive surgical margins, and/or high CAPRA-S score (score for prediction of post-operative BCR)), although this was only statistically significant for pathological T-stage and CAPRA-S score (Additional file 9). Consistent with this, patients with low *MEIS2* methylation had significantly lower CAPRA-S score (*p* = 0.0066 and 0.0419, respectively; Mann-Whitney test; Fig. 6). Combining the two qMSP assays into one model (low methylation in both assays vs. high methylation in at least one assay), low methylation was still significantly associated with low CAPRA-S score (*p* = 0.0004; Mann-Whitney test. Fig. 6).

**Fig. 7:**
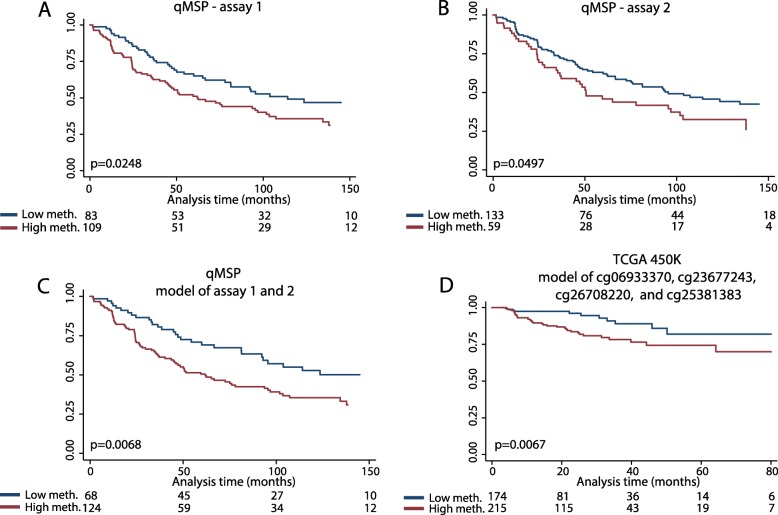
Kaplan-Meier survival estimates of *MEIS2* DNA methylation using time to BCR as endpoint. **a** BCR-free survival curve for assay 1 in the qMSP cohort. **b** BCR-free survival curve for assay 2 in the qMSP cohort. **c** BCR-free survival curve for assay 1 and 2 combined (model) in the qMSP cohort. **d** BCR-free survival curve for the MEIS2 methylation model of cg06933370, cg23677243, cg26708220, and cg25381383 in TCGA 450K data. ***p*** values were calculated using the log-rank test. Meth., methylation

Furthermore, for each assay, high *MEIS2* methylation was significantly associated with short BCR-free survival in Kaplan-Meier analysis (qMSP assay 1/2, *p* = 0.0248/*p* = 0.0497; log-rank test; Fig. 7a, b). Similar results were obtained for the individual qMSP assays by univariate cox regression analysis but was only borderline significant for assay 2 (assay 1, *p* = 0.026, HR = 1.57 (1.06–2.34), C-index = 0.564; assay 2, *p* = 0.051, HR = 1.49 (1.00-2.22), C-index = 0.546; Additional files 10 and 11). However, the prognostic power was improved by combining the two assays, as low methylation for both assays (compared to high methylation for at least one of the assays) was associated with significantly better post-operative BCR-free survival in both Kaplan-Meier (*p* = 0.0068, log-rank test; Fig. 7c) and univariate cox regression analysis (*p* = 0.008, HR = 1.79 (1.17–2.76), C-index = 0.573; Table 2). However, the combined *MEIS2* methylation model did not remain significant after adjustment for routine clinicopathological variables (*p* = 0.681, HR = 1.10 (0.69–1.75), c-index = 0.730, multivariate cox regression; Table 2).

**Table 2:** Uni- and multivariate cox regression of the *MEIS2* methylation model in RP patients analyzed by qMSP (*n* = 192)

Variable		Univariate			Multivariate		
		HR (CI)	*p* value	C-index	HR (CI)	*p* value	C-index
*MEIS2* model meth.	Low vs. high	1.79 (1.17–2.76)	0.008	0.573	1.10 (0.69–1.75)	0.681	0.730
Path. Gleason Score	< 7	Ref		0.647	Ref		
	= 7	2.79 (1.63–4.76)	0.000		2.91 (1.67–5.07)	0.000	
	> 7	5.28 (2.92–9.56)	0.000		4.22 (2.26–7.87)	0.000	
Path. T-stage	T2 vs. T3	3.16 (2.13–4.68)	0.000	0.634	2.22 (1.33–3.68)	0.002	
Surgical margin status	Neg vs. pos.	2.91 (1.96–4.33)	0.000	0.626	1.50 (0.90–2.51)	0.120	
Path. N-stage	pN0 vs. pN1	0.53 (0.22–1.31)	0.169	0.523	–	–	–

BCR was used as endpoint. *Meth.* methylation*, Path.* pathologic*, HR* hazard ratio*, CI* confidence interval

Next, for external validation, we used the PC patient cohort from TCGA (450K data; assay 1, average methylation of cg06933370, cg23677243, and cg26708220; assay 2, methylation of cg25381383). In this cohort, high *MEIS2* DNA methylation was generally associated with adverse clinicopathological parameters although only significant for probes corresponding to assay 1 regarding pathological T-stage and for probes corresponding to assay 2 regarding both Gleason score and pathological T-stage (Additional file 5). Moreover, low *MEIS2* DNA methylation was associated with significantly better post-operative BCR-free survival in uni- and multivariate analysis ( “Assay 1”/”Assay 2”, *p* = 0.024/0.017, HR = 1.98 (1.09–3.59)/2.09 (1.14–3.82), C-index = 0.712/0.717, multivariate cox regression. Additional files 12 and 13).

Likewise, low methylation of the combined model was associated with significantly better BCR-free survival also in the TCGA cohort by both Kaplan-Meier (*p* = 0.0067, Log-rank test; Fig. 7d) and univariate cox regression analysis (*p* = 0.009, HR = 2.48 (1.26–4.88); Table 3). Furthermore, after adjusting for pathological Gleason score and T-stage, the combined *MEIS2* methylation model remained a significant independent predictor of BCR (*p* = 0.042, HR = 2.02 (1.02–3.99); Table 3). In summary, this study is the first to demonstrate a significant association between aberrant *MEIS2* hypermethylation and adverse clinical outcome of PC.

**Table 3:** Uni- and multivariate cox regression of the *MEIS2* methylation model in the 450K TCGA cohort (*n* = 389)

Variable		Univariate			Multivariate		
		HR (CI)	*p* value	C-index	HR (CI)	*p* value	C-index
*MEIS2* model meth.	Low vs. high	2.48 (1.26–4.88)	0.009	0.618	2.02 (1.02–3.99)	0.042	0.710
Gleason score	< 7	1		0.646	1		
	= 7	2.77 (0.63–12.13)	0.176		1.64 (0.37–7.27)	0.517	
	> 7	7.53 (1.80–31.58)	0.006		3.63 (0.84–15.69)	0.085	
Path. T-stage	T2 vs. T3	6.02 (2.16–16.81)	0.001	0.617	3.97 (1.38–11.43)	0.011	
Surgical margin status	Neg. vs. pos.	1.47 (0.82–2.65)	0.198	0.542	–	–	–

BCR was used as end-point. *Meth.* methylation, *Path.* pathologic, *HR* hazard ratio, *CI* confidence interval

## Discussion

In this study, we performed an integrative analysis of DNA methylation and RNA expression data from PC, as this approach may identify novel candidate driver genes involved in PC development or progression. Accordingly, we conducted DNA methylation profiling (450K array) and RNAseq on PC and AN samples from 29 radical prostatectomy patients and integrated the data for biomarker discovery. Among the top candidate genes with significantly altered DNA methylation and/or RNA expression levels in PC, we found significant enrichment for homeobox genes, incl. *MEIS2.* RNA expression and DNA methylation of MEIS2 were inversely correlated in our discovery cohort, which was confirmed in a large independent RP patient cohort from TCGA (495 PC, 36 AN), suggesting epigenetic silencing. Furthermore, low transcriptional expression and DNA hypermethylation of *MEIS2* was associated with post-operative BCR in multiple independent RP patient cohorts, including more than 700 PC patients in total. To the best of our knowledge, this is the first study to demonstrate significant prognostic value of *MEIS2* epigenetic silencing in PC.

The present study is the first to examine the prognostic potential of MEIS2 transcriptional expression in PC. We used three public RNA expression dataset from RP cohorts (> 700 patients with clinical follow-up) and found that low MEIS2 expression was associated with post-operative BCR and generally correlated with adverse clinicopathological parameters. After adjustment for routine clinicopathological variables, MEIS2 expression remained a significant predictor of BCR in multivariate analysis in the Long cohort, but not in the TCGA or Taylor cohort, possible due to differences in the exact composition of the cohorts and/or in the methodologies used for expression profiling. Specifically, the Long RNAseq data was derived from FFPE tissue samples, whereas Taylor et al. used microarrays and TCGA used fresh frozen tissue samples for RNAseq analyses. Furthermore, the fraction of patients with BCR varied from 12% in TCGA to 25% in the Taylor cohort and 52% in the Long cohort, possibly affecting the statistical power. Nevertheless, our results showed that low MEIS2 transcriptional expression was associated with significantly shorter BCR-free survival in all three cohorts, which corroborates and expands on previous findings of a significant association between MEIS2 protein expression and short overall survival in PC [16].

We found a significant inverse correlation between MEIS2 DNA methylation and RNA expression, suggesting that *MEIS2* is epigenetically silenced in PC. The observed silencing of *MEIS2* could also be affected by other factors, e.g., transcriptional regulators expressed during PC development/progression, but further studies are needed to evaluate this. While our finding of frequent aberrant hypermethylation of *MEIS2* in PC is consistent with results from two previous studies exploring the methylome in PC [36, 37], our demonstration of diagnostic and prognostic potential of *MEIS2* DNA methylation has not been described before. Here, we found that *MEIS2* hypermethylation had diagnostic potential for PC with a high AUC (AUC assay 1/2, 0.814/0.917), comparable to AUCs reported for previously published candidate methylation markers for PC (AUCs 0.794–0.980) [5, 6, 8, 9, 11, 38]. Some of these previously published DNA methylation markers could predict BCR after RP independent of routine clinicopathological parameters [5, 6]; however, this was only the case for *MEIS2* in the 450K data from TCGA and not in the qMSP cohort. In addition, we found that high *MEIS2* methylation levels were significantly associated with short BCR-free survival and high CAPRA-S score. *MEIS2* methylation also predicted time to BCR independently of clinicopathological parameters in our external validation cohort from TCGA. However, the prognostic potential of *MEIS2* DNA methylation warrants further validation in a large cohort with long clinical follow-up as the TCGA cohort used for independent validation had short clinical follow-up and few events (BCR in 12% of patients). In this study, we made a prognostic model based on *MEIS2* methylation. Conceivably, adding RNA expression data to this model may potentially improve predictive accuracy, but we found no significant evidence for this in the TCGA cohort (data not shown). However, further studies on large cohorts with overlapping DNA methylation and RNA expression data and long follow-up are needed to investigate this.

MEIS2 RNA and protein expression has previously been reported as downregulated in primary PC and further downregulated in metastatic PC tissue, as compared to non-malignant prostate tissue samples [16, 17]. Furthermore, in a small cohort of 83 Gleason 6 patients, a low protein level of MEIS2 was significantly associated with short overall survival [16]. Also, downregulation of MEIS2 has been reported to play an important functional role in progression to castration resistant PC [18]. This corresponds with our findings of low MEIS2 transcriptional expression being associated with more aggressive PC, defined by short BCR-free survival. Although epigenetic silencing of *MEIS2* has also been described in lung and hepatocellular carcinoma cell lines as well as colorectal cancer [39–41], the function of *MEIS2* in other cancers remains unclear and may be highly disease-specific as *MEIS2* is downregulated in some cancers and upregulated in others [13]. In neuroblastoma, leukemia, and multiple myeloma, *MEIS2* serves as an oncogene [42–45] and, similarly seems to play an important role in tumor cell migration and invasion in bladder and colorectal cancer [46, 47]. *MEIS2* expression may also be involved in chemotherapy sensitivity, although current results are conflicting. Thus, MEIS2 knockdown increases responsiveness to chemotherapy in multiple myeloma, whereas MEIS2 is downregulated in colorectal cancer patients resistant towards oxaliplatin-based chemotherapy [41, 45]. Contradictory results also exist regarding prognosis. In ovarian cancer, high MEIS2 protein expression has been associated with improved prognosis whereas high RNA expression of MEIS2 has been associated with worse overall survival in colorectal cancer [47, 48]. Thus, the functional role of MEIS2 seems to be highly disease-specific and further studies are needed to clarify this. In the present study, we examined the prognostic potential of MEIS2 DNA methylation and transcriptional expression in five RP cohorts. Risk stratification of PC patients after RP is important to identify patients who would benefit from adjuvant therapy and to avoid treating patients with low risk of BCR. In this setting, a prognostic molecular marker as *MEIS2* could improve the current management of RP patients. Additionally, it is also of clinical relevance to test the prognostic value of *MEIS2* in diagnostic biopsies, as this could give important clues to whether MEIS2 DNA methylation and/or RNA expression can improve the accuracy of PC prognosis at the time of diagnosis. Currently, only pre-operative clinicopathological parameters are available at diagnosis which are upgraded and/or upstaged after RP in more than 50% of PC tumors [49]. Thus, improved prediction of aggressiveness at diagnosis by addition of a molecular marker could improve treatment decisions and reduce overtreatment of indolent PCs.

A potential limitation to our study is that we could not discriminate between Gleason scores 3+4 and 4+3, as this information was not available for the public PC patient sets. Moreover, we have used BCR as endpoint in our survival analyses. BCR is only a surrogate marker for aggressiveness and more clinically relevant endpoints as metastatic disease or PC-specific mortality should be used in future studies instead. However, this would require > 15 years of clinical follow-up due to the generally slow natural history of PC progression [50].

## Conclusions

In conclusion, we here show that the homeobox gene *MEIS2* is epigenetically silenced in PC. To the best of our knowledge, this is the first study to investigate, demonstrate, and independently validate a prognostic biomarker potential for MEIS2 at the transcriptional expression level and at the DNA methylation level in PC.

## Supplementary information

**Additional file 1: Table S1.** Clinicopathological characteristics of the discovery cohort.

**Additional file 2: Table S2.** Primers and probes used for qMSP. For each probe, 5’ fluorophores and 3’ quenchers are given.

**Additional file 3: Figure S1**. MDS plots of PC and AN samples from the discovery cohort. For the 450K data, the 1000 most variable CpG sites were used, whereas the 150 most variable genes were used for the RNAseq data.

**Additional file 4: Figure S2.** Functional clustering of differentially methylated and expressed genes (discovery cohort) using DAVID. For the 450K data, the 3000 genes with the lowest BH-adjusted p-value (PC vs. AN) were used for the analysis. Top 10 enriched clusters are shown. For the RNAseq data, the 2314 genes with a significant BH-adjusted p-value (PC vs. AN) were used as input. Top 10 enriched clusters as well as cluster number 51 (homeobox-cluster) are shown. Barplots show enrichment scores of the clusters. A general term describing the genes/categories within each cluster is given.

**Additional file 5: Figure S3**. Association between RNA expression and DNA methylation of MEIS2 in public cohorts. Clinicopathological variables examined: pathological Gleason score, pathological T-stage, and surgical margin status. Public cohorts examined: Long et al. (RNAseq), Taylor (microarray), TCGA (RNAseq and 450K). P-values were calculated using Mann-Whitney tests. Path., pathological. Surg., surgical. P, p-value.

**Additional file 6: Table S3.** Uni- and multivariate cox regression of MEIS2 RNA expression in the Long RNAseq cohort. BCR was used as end-point (n=106 patients). Cont.: continuous. Path.: Pathologic. HR: Hazard ratio. CI: Confidence interval. BCR, biochemical recurrence.

**Additional file 7: Table S4.** Uni- and multivariate cox regression of MEIS2 RNA expression in the Taylor microarray cohort. BCR was used as end-point (n=126 patients). Cont.: continuous. Path.: Pathologic. HR: Hazard ratio. CI: Confidence interval. BCR, biochemical recurrence.

**Additional file 8: Table S5.** Uni- and multivariate cox regression of MEIS2 RNA expression in the TCGA RNAseq cohort. BCR was used as end-point (n=389 patients). Cont.: continuous. Path.: Pathologic. HR: Hazard ratio. CI: Confidence interval. BCR, biochemical recurrence.

**Additional file 9: Figure S4**. Association between DNA methylation of MEIS2 assay 1 and 2 and clinicopathological variables in the qMSP cohort. Clinicopathological variables examined: pathological Gleason score, pathological T-stage, surgical margin status, and CAPRA-S score (low: 0-2, intermediate: 3-5, high: ≥6). P-values were calculated using Mann-Whitney tests. Path., pathological. Surg., surgical. P, p-value.

**Additional file 10: Table S6.** Uni- and multivariate cox regression of MEIS2 assay 1 in the qMSP cohort using BCR as end-point (n=195 patients). Meth.: Methylation. Path.: Pathologic. HR: Hazard ratio. CI: Confidence interval.

**Additional file 11: Table S7.** Uni- and multivariate cox regression of MEIS2 assay 2 in the qMSP cohort using BCR as end-point (n=195 patients). Meth.: Methylation. Path.: Pathologic. HR: Hazard ratio. CI: Confidence interval.

**Additional file 12: Table S8.** Uni- and multivariate cox regression of the average β-value of cg06933370, cg23677243, and cg26708220 (to mimic qMSP assay 1) in the TCGA 450K cohort (n=389 patients). BCR was used as end-point. Meth: Methylation. Path.: Pathologic. HR: Hazard ration. CI: Confidence interval.

**Additional file 13: Table S9.** Uni- and multivariate cox regression of cg25381383 in the TCGA 450K cohort (n=389 patients). BCR was used as end-point. Meth: Methylation. Path.: Pathologic. HR: Hazard ration. CI: Confidence interval.

## Data Availability

The data generated in this study is available from the corresponding author upon reasonable request. The RNAseq, 450K, and clinical data from TCGA are available at the Cancer Genome Atlas (http://cancergenome.nih.gov/) [29]. From Gene Expression Omnibus (GEO, https://www.ncbi.nlm.nih.gov/geo/query/acc.cgi?acc=GSE21034), the RNAseq and clinical data from Long et al. (GSE54460) [28] as well as the microarray and clinical data from Taylor et al. (GSE21034) [35] are available.

## Abbreviations

450K: Illumina 450K DNA methylation array
AN: Adjacent normal
AUC: Area under the curve
BCR: Biochemical recurrence
BH: Benjamini-Hochberg
BPH: Benign prostatic hyperplasia
CI: Confidence interval
CPM: Counts per million
CRPC: Castration resistant prostate cancer
DAVID: Database for Annotation, Visualization and Integrated Discovery
FFPE: Formalin-fixed paraffin-embedded
HE: Hematoxylin and eosin
HR: Hazard ratio
MDS: Multi-dimensional scaling
Path.: Pathological
PC: Prostate cancer
PSA: Prostate-specific antigen
qMSP: Quantitative methylation specific PCR
RNAseq: RNA sequencing
ROC: Receiver operating characteristics
RP: Radical prostatectomy
TALE: Three amino acid loop extensions
TCGA: The Cancer Genome Atlas
TURP: Transurethral resection of the prostate
WGA: Whole-genome amplified
