# Supplementary material for: Epigenetic silencing of MEIS2 in prostate cancer recurrence
Source: Clin Epigenetics. 2019 Oct 22;11:147. doi: 10.1186/s13148-019-0742-x (PMC6805635; doi:10.1186/s13148-019-0742-x)
Supplement: Supplementary file 1 — Additional file 1: Table S1. Clinicopathological characteristics of the discovery cohort. [file 13148_2019_742_MOESM1_ESM.docx]

Additional file 1: Table S1

Clinicopathological characteristics of the discovery cohort*.*

| **Variable** | **PC (n=29)** |
| --- | --- |
| **Age at RP, median (range)** | 64 (48-73) |
| **Pre-operative PSA (ng/mL), median (range)** | 16.2 (4-62.1) |
| **Pathological Gleason score** |  |
| **≤6, n (%)** | 8 (27.6) |
| **=7, n (%)** | 14 (48.3) |
| **≥8, n (%)** | 7 (24.1) |
| **Pathological T-stage** |  |
| **≤pT2c, n (%)** | 6 (20.7) |
| **≥pT3a, n (%)** | 23 (79.3) |
| **Surgical margin status** |  |
| **Cancer negative margin, n (%)** | 20 (69) |
| **Cancer positive margin, n (%)** | 9 (31) |
| **Follow-up** |  |
| **No follow-up, n (%)** | 14 (48.3) |
| **Follow-up, n (%)** | 15 (51.7) |
| **Follow-up (months), median (range)** | 55.5 (2-130) |
| **PSA recurrence** |  |
| **No follow-up, n (%)** | 14 (48.3) |
| **No PSA recurrence, n (%)** | 6 (20.7) |
| **PSA recurrence, n (%)** | 9 (31.0) |
|  | |
|  | **AN (n=15)** |
| **Age at RP, median (range)** | 61 (54-73) |
