# Supplementary material for: Epigenetic silencing of MEIS2 in prostate cancer recurrence
Source: Clin Epigenetics. 2019 Oct 22;11:147. doi: 10.1186/s13148-019-0742-x (PMC6805635; doi:10.1186/s13148-019-0742-x)
Supplement: Supplementary file 2 — Additional file 2: Table S2. Primers and probes used for qMSP. For each probe, 5’ fluorophores and 3’ quenchers are given. [file 13148_2019_742_MOESM2_ESM.docx]

Additional file 2: Table S2

Primers and probes used for qMSP.

| **Assay** |  | **Sequence (5’-3’)** |
| --- | --- | --- |
| **MEIS2 assay 1** | Forward primer | AGTTTATTTTTGGGGTCGAG |
|  | Reverse primer | GCGATAAAAAACGAAACCC |
|  | Probe | FAM-TTTCGGCGTTCGGTTTTTCG-BHQ1 |
| **MEIS2 assay 2** | Forward primer | GCGTTTTTGTTAGTTTTTTCG |
|  | Reverse primer | AACCATCATAACAATAATATCTACGA |
|  | Probe | FAM-TTATTTTTTTTTATATGTGAGGGTCGATTT-BHQ1 |
| **aluC4** | Forward primer | GGTTAGGTATAGTGGTTTATATTTGTAATTTTAGTA |
|  | Reverse primer | ATTAACTAAACTAATCTTAAACTCCTAACCTCA |
|  | Probe | FAM-CCTACCTTAACCTCCC-MGBNFQ |
| **MYOD1** | Forward primer | CCAACTCCAAATCCCCTCTCTAT |
|  | Reverse primer | TGGTTTTTTTAGGGAGTAAGTTTGTT |
|  | Probe | FAM-TCCCTTCCTATTCCTAAATCCAACCTAAATACCTCC-BHQ1 |

*For each probe, 5’ flurophors and 3’ quenchers are given.*
