# Supplementary figures and images for: Epigenetic silencing of MEIS2 in prostate cancer recurrence
Source: Clin Epigenetics. 2019 Oct 22;11:147. doi: 10.1186/s13148-019-0742-x (PMC6805635; doi:10.1186/s13148-019-0742-x)

Figure S1

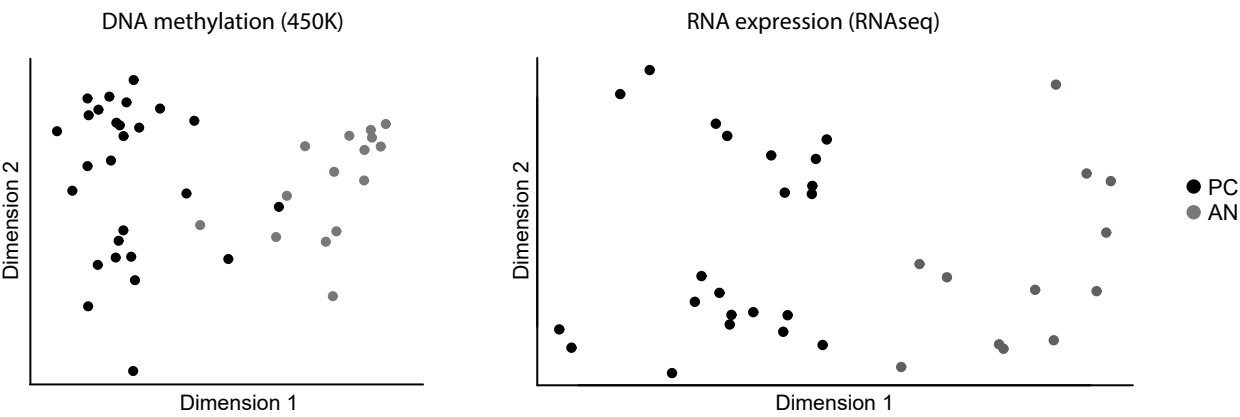

Supplement: Supplementary file 3 — Additional file 3: Figure S1. MDS plots of PC and AN samples from the discovery cohort. For the 450K data, the 1000 most variable CpG sites were used, whereas the 150 most variable genes were used for the RNAseq data. [file 13148_2019_742_MOESM3_ESM.pdf]

Figure S2

DAVID Functional Annotation clusters

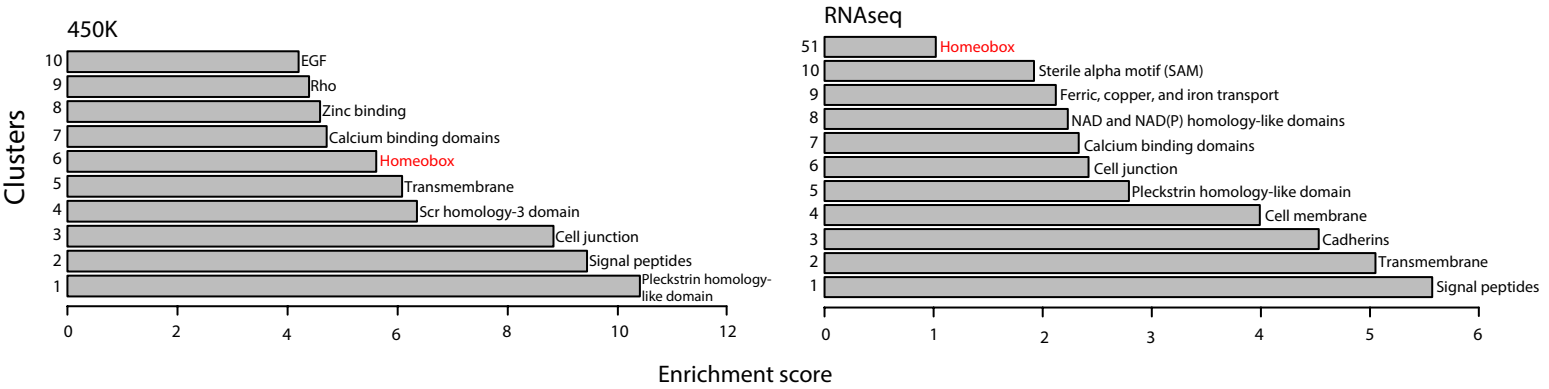

Supplement: Supplementary file 4 — Additional file 4: Figure S2. Functional clustering of differentially methylated and expressed genes (discovery cohort) using DAVID. For the 450K data, the 3000 genes with the lowest BH-adjusted p-value (PC vs. AN) were used for the analysis. Top 10 enriched clusters are shown. For the RNAseq data, the 2314 genes with a significant BH-adjusted p-value (PC vs. AN) were used as input. Top 10 enriched clusters as well as cluster number 51 (homeobox-cluster) are shown. Barplots show enrichment scores of the clusters. A general term describing the genes/categories within each cluster is given. [file 13148_2019_742_MOESM4_ESM.pdf]

Figure S3

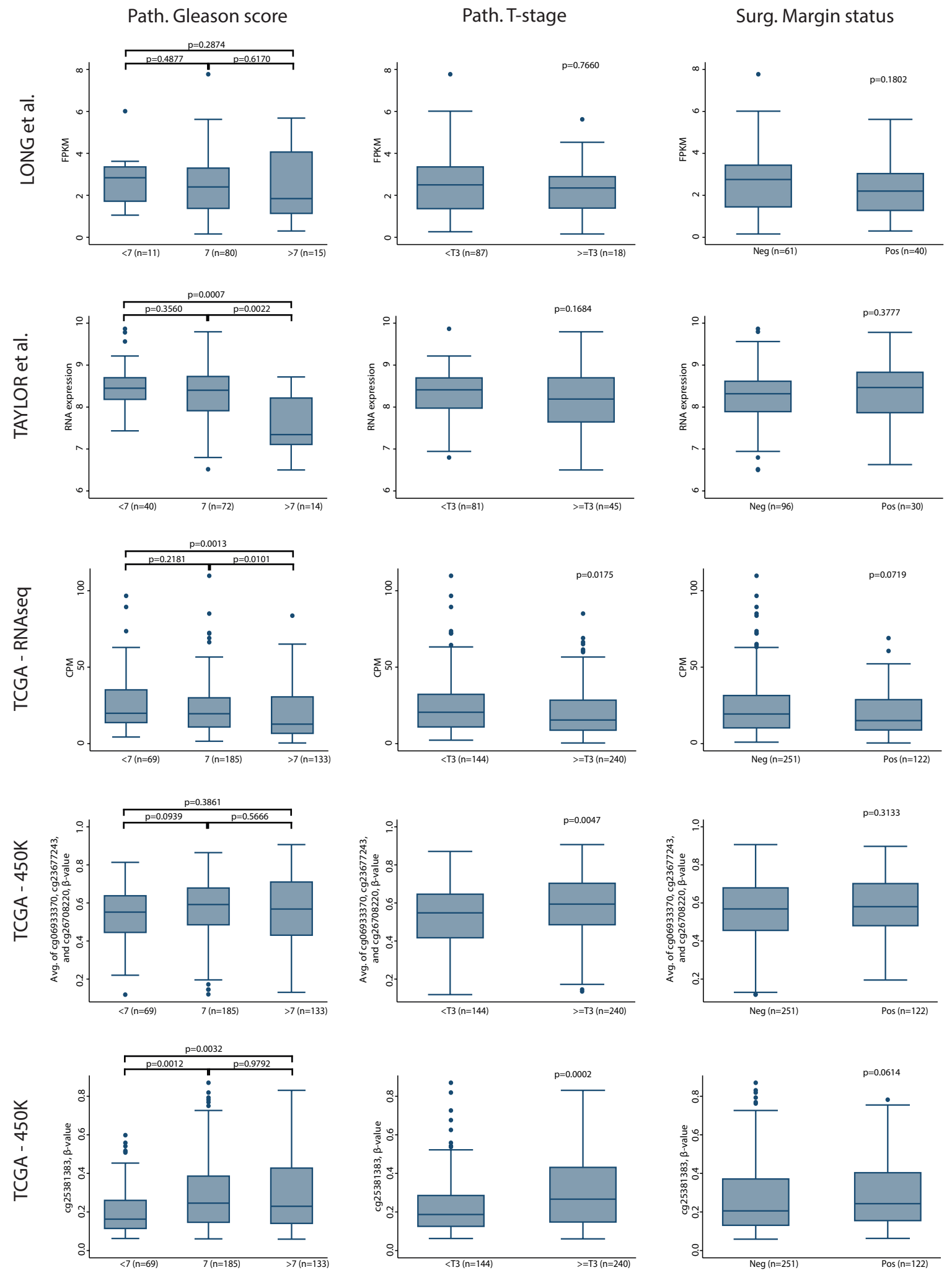

Supplement: Supplementary file 5 — Additional file 5: Figure S3. Association between RNA expression and DNA methylation of MEIS2 in public cohorts. Clinicopathological variables examined: pathological Gleason score, pathological T-stage, and surgical margin status. Public cohorts examined: Long et al. (RNAseq), Taylor (microarray), TCGA (RNAseq and 450K). P-values were calculated using Mann-Whitney tests. Path., pathological. Surg., surgical. P, p-value. [file 13148_2019_742_MOESM5_ESM.pdf]

Figure S4

MEIS2 ssay 1

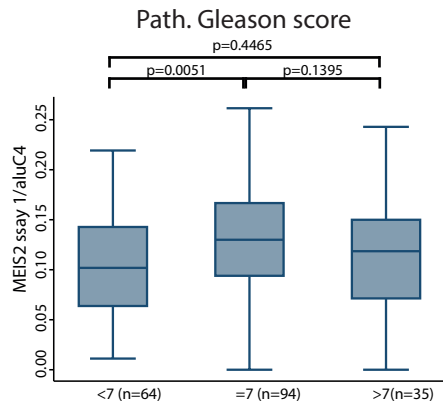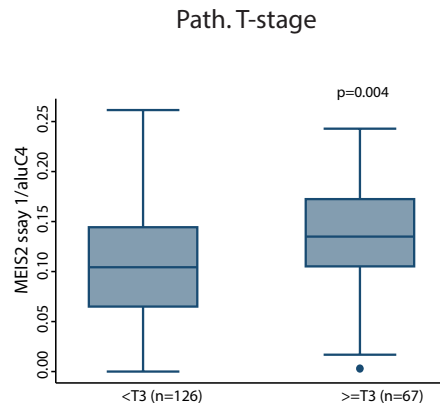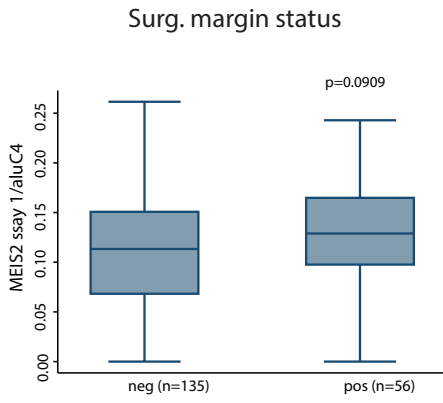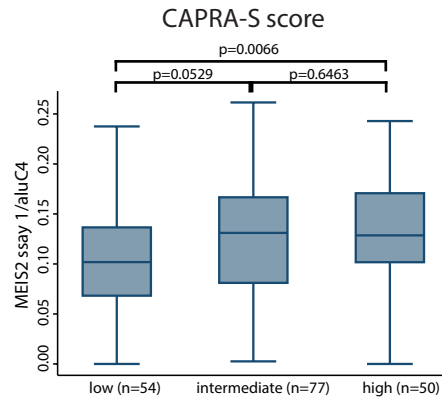

MEIS2 ssay 2

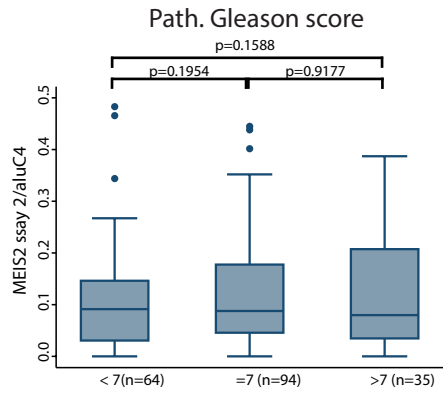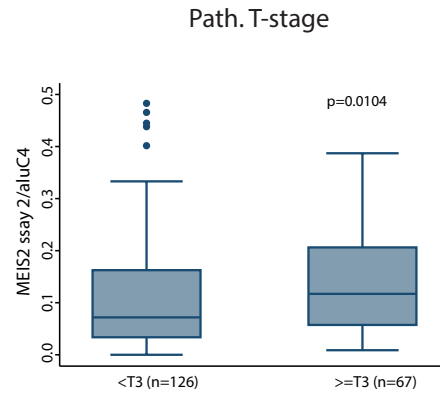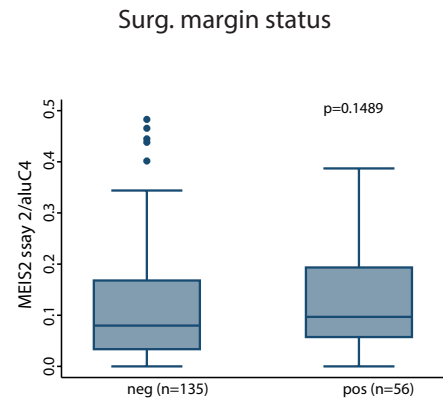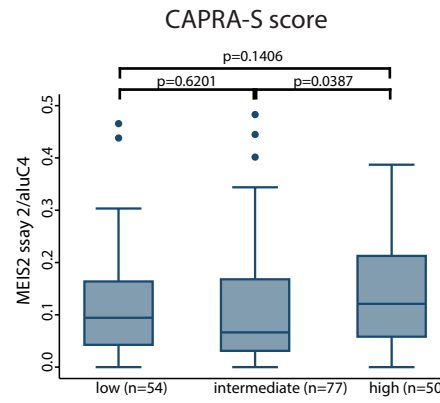

Supplement: Supplementary file 9 — Additional file 9: Figure S4. Association between DNA methylation of MEIS2 assay 1 and 2 and clinicopathological variables in the qMSP cohort. Clinicopathological variables examined: pathological Gleason score, pathological T-stage, surgical margin status, and CAPRA-S score (low: 0-2, intermediate: 3-5, high: ≥6). P-values were calculated using Mann-Whitney tests. Path., pathological. Surg., surgical. P, p-value. [file 13148_2019_742_MOESM9_ESM.pdf]
