# Supplementary material for: Epigenetic silencing of MEIS2 in prostate cancer recurrence
Source: Clin Epigenetics. 2019 Oct 22;11:147. doi: 10.1186/s13148-019-0742-x (PMC6805635; doi:10.1186/s13148-019-0742-x)
Supplement: Supplementary file 6 — Additional file 6: Table S3. Uni- and multivariate cox regression of MEIS2 RNA expression in the Long RNAseq cohort. BCR was used as end-point (n=106 patients). Cont.: continuous. Path.: Pathologic. HR: Hazard ratio. CI: Confidence interval. BCR, biochemical recurrence. [file 13148_2019_742_MOESM6_ESM.docx]

Additional file 6: Table S3

Uni- and multivariate cox regression of MEIS2 RNA expression in the Long RNAseq cohort.

| **Variable** | | **Univariate** | | | **Multivariate** | | |
| --- | --- | --- | --- | --- | --- | --- | --- |
|  |  | **HR (CI)** | **p-val** | **C-index** | **HR (CI)** | **p-val** | **C-index** |
| **Continuous expression** | | | | | | | |
| **MEIS2 RNA expression** | Cont. | 0.75 (0.59-0.96) | 0.020 | 0.614 | 0.67 (0.50-0.88) | 0.004 | 0.784 |
| **Path. Gleason score** | <7 | 1 | | 0.584 | 1 | |  |
|  | 7 | 1.35 (0.48-3.82) | 0.570 |  | 1.28 (0.38-4.33) | 0.691 |  |
|  | >7 | 3.34 (1.03-10.87) | 0.045 |  | 2.16 (0.51-9.24) | 0.299 |  |
| **PSA** | Cont. | 1.09 (1.06-1.13) | 0.000 | 0.699 | 1.09 (1.05-1.13) | 0.000 |  |
| **Surgical margin status** | Pos vs. neg | 3.16 (1.72-5.80) | 0.000 | 0.643 | 2.28 (1.18-4.41) | 0.014 |  |
| **Path. T-stage** | T2 vs. T3 | 1.55 (0.74-3.22) | 0.245 | 0.539 | - | - | - |
| **Dichotomized expression** | | | | | | | |
| **MEIS2 RNA expression** | Low vs. high | 0.46 (0.26-0.83) | 0.010 | 0.585 | 0.39 (0.20-0.76) | 0.005 | 0.757 |
| **Path. Gleason score** | <7 | 1 | | 0.584 | 1 | |  |
|  | 7 | 1.35 (0.48-3.82) | 0.570 |  | 1.49 (0.44-5.04) | 0.522 |  |
|  | >7 | 3.34 (1.03-10.87) | 0.045 |  | 1.72 (0.39-7.64) | 0.474 |  |
| **PSA** | Cont. | 1.09 (1.06-1.13) | 0.000 | 0.699 | 1.10 (1.05-1.13) | 0.000 |  |
| **Surgical margin status** | Pos vs. neg | 3.16 (1.72-5.80) | 0.000 | 0.643 | 2.51 (1.32-4.79) | 0.005 |  |
| **Path. T-stage** | T2 vs. T3 | 1.55 (0.74-3.22) | 0.245 | 0.539 | - | - | - |

*BCR was used as end-point (n=106 patients). Cont.: continuous. Path.: Pathologic. HR: Hazard ratio. CI: Confidence interval. BCR, biochemical recurrence*
