# Supplementary material for: Epigenetic silencing of MEIS2 in prostate cancer recurrence
Source: Clin Epigenetics. 2019 Oct 22;11:147. doi: 10.1186/s13148-019-0742-x (PMC6805635; doi:10.1186/s13148-019-0742-x)
Supplement: Supplementary file 7 — Additional file 7: Table S4. Uni- and multivariate cox regression of MEIS2 RNA expression in the Taylor microarray cohort. BCR was used as end-point (n=126 patients). Cont.: continuous. Path.: Pathologic. HR: Hazard ratio. CI: Confidence interval. BCR, biochemical recurrence. [file 13148_2019_742_MOESM7_ESM.docx]

Additional file 7: Table S4

Uni- and multivariate cox regression of MEIS2 RNA expression in the Taylor microarray cohort.

| **Variable** | | **Univariate** | | | **Multivariate** | | |
| --- | --- | --- | --- | --- | --- | --- | --- |
|  |  | **HR (CI)** | **p-val** | **C-index** | **HR (CI)** | **p-val** | **C-index** |
| **Continuous expression** | | | | | | | |
| **MEIS2 RNA expression** | Cont. | 0.50 (0.28-0.90) | 0.020 | 0.598 | 0.88 (0.51-1.53) | 0.652 | 0.809 |
| **Path. Gleason Score** | <7 | 1 | | 0.769 | 1 | |  |
|  | =7 | 3.69 (1.08-12.60) | 0.037 |  | 2.96 (0.85-10.29) | 0.087 |  |
|  | >7 | 24.83 (6.90-89.30) | 0.000 |  | 15.08 (3.87-58.71) | 0.000 |  |
| **PSA** | Cont. | 1.02 (1.01-1.04) | 0.000 | 0.645 | 1.02 (1.00-1.04) | 0.027 |  |
| **Path. T-stage** | T2 vs. T3 | 4.12 (2.01-8.44) | 0.000 | 0.694 | 2.72 (1.25-5.91) | 0.011 |  |
| **Dichotomized expression** | | | | | | | |
| **MEIS2 RNA expression** | Low vs. high | 0.25 (0.11-0.54) | 0.000 | 0.617 | 0.63 (0.25-1.59) | 0.331 | 0.816 |
| **Path. Gleason Score** | <7 | 1 | | 0.769 | 1 | |  |
|  | =7 | 3.69 (1.08-12.60) | 0.037 |  | 3.01 (0.87-10.43) | 0.083 |  |
|  | >7 | 24.83 (6.90-89.30) | 0.000 |  | 14.02 (3.63-54.20) | 0.000 |  |
| **PSA** | Cont. | 1.02 (1.01-1.04) | 0.000 | 0.645 | 1.01 (1.00-1.04) | 0.035 |  |
| **Path. T-stage** | T2 vs. T3 | 4.12 (2.01-8.44) | 0.000 | 0.694 | 2.65 (1.22-5.79) | 0.014 |  |

*BCR was used as end-point (n=126 patients). Cont.: continuous. Path.: Pathologic. HR: Hazard ratio. CI: Confidence interval. BCR, biochemical recurrence.*
