# Supplementary material for: Epigenetic silencing of MEIS2 in prostate cancer recurrence
Source: Clin Epigenetics. 2019 Oct 22;11:147. doi: 10.1186/s13148-019-0742-x (PMC6805635; doi:10.1186/s13148-019-0742-x)
Supplement: Supplementary file 8 — Additional file 8: Table S5. Uni- and multivariate cox regression of MEIS2 RNA expression in the TCGA RNAseq cohort. BCR was used as end-point (n=389 patients). Cont.: continuous. Path.: Pathologic. HR: Hazard ratio. CI: Confidence interval. BCR, biochemical recurrence. [file 13148_2019_742_MOESM8_ESM.docx]

Additional file 8: Table S5

*Uni- and multivariate cox regression of MEIS2 RNA expression in the TCGA RNAseq cohort.*

| **Variable** | | **Univariate** | | | **Multivariate** | | |
| --- | --- | --- | --- | --- | --- | --- | --- |
|  |  | **HR (CI)** | **p-val** | **C-index** | **HR (CI)** | **p-val** | **C-index** |
| **Continuous expression** | | | | | | | |
| **MEIS2 RNA expression** | Cont. | 0.99 (0.97-1.01) | 0.301 | 0.556 | 1.00 (0.98-1.02) | 0.816 | 0.685 |
| **Gleason score** | <7 | 1 | | 0.646 | 1 | |  |
|  | =7 | 2.77 (0.63-12.13) | 0.176 |  | 1.80 (0.40-8.01) | 0.443 |  |
|  | >7 | 7.53 (1.80-31.58) | 0.006 |  | 3.93 (0.90-17.11) | 0.068 |  |
| **Path. T-stage** | T2 vs. T3 | 6.02 (2.16-16.81) | 0.001 | 0.617 | 4.54 (1.59-12.98) | 0.005 |  |
| **Surgical margin status** | Neg. vs. pos. | 1.47 (0.82-2.65) | 0.198 | 0.542 | - | - | - |
| **Dichotomized expression** | | | | | | | |
| **MEIS2 RNA expression** | Low vs. high | 0.50 (0.28-0.90) | 0.022 | 0.593 | 0.67 (0.36-1.24) | 0.201 | 0.694 |
| **Gleason score** | <7 | 1 | | 0.646 | 1 | |  |
|  | =7 | 2.77 (0.63-12.13) | 0.176 |  | 1.83 (0.41-8.16) | 0.426 |  |
|  | >7 | 7.53 (1.80-31.58) | 0.006 |  | 3.57 (0.82-15.66) | 0.091 |  |
| **Path. T-stage** | T2 vs. T3 | 6.02 (2.16-16.81) | 0.001 | 0.617 | 4.20 (1.45-12.3) | 0.008 |  |
| **Surgical margin status** | Neg. vs. pos. | 1.47 (0.82-2.65) | 0.198 | 0.542 | - | - | - |

*BCR was used as end-point (n=389 patients). Cont.: continuous. Path.: Pathologic. HR: Hazard ratio. CI: Confidence interval. BCR, biochemical recurrence.*
