# Supplementary material for: Epigenetic silencing of MEIS2 in prostate cancer recurrence
Source: Clin Epigenetics. 2019 Oct 22;11:147. doi: 10.1186/s13148-019-0742-x (PMC6805635; doi:10.1186/s13148-019-0742-x)
Supplement: Supplementary file 11 — Additional file 11: Table S7. Uni- and multivariate cox regression of MEIS2 assay 2 in the qMSP cohort using BCR as end-point (n=195 patients). Meth.: Methylation. Path.: Pathologic. HR: Hazard ratio. CI: Confidence interval. [file 13148_2019_742_MOESM11_ESM.docx]

Additional file 11: **Table S7**

*Uni- and multivariate cox regression of MEIS2 assay 2 in the qMSP cohort using BCR as end-point (n=195 patients).*

| Variable | | Univariate | | | Multivariate | | |
| --- | --- | --- | --- | --- | --- | --- | --- |
|  |  | HR (CI) | p-val | C-index | HR (CI) | p-val | C-index |
| *MEIS2* assay 2 meth. | Low vs. high | 1.49 (1.00-2.22) | 0.051 | 0.546 | 1.07 (0.70-1.62) | 0.756 | 0.730 |
| Path. Gleason Score | <7 | 1 | | 0.647 | 1 | |  |
|  | =7 | 2.79 (1.63-4.76) | 0.000 |  | 2.93 (1.68-5.09) | 0.000 |  |
|  | >7 | 5.28 (2.92-9.56) | 0.000 |  | 4.20 (2.23-7.92) | 0.000 |  |
| Path. T-stage | T2 vs. T3 | 3.16 (2.13-4.68) | 0.000 | 0.634 | 2.24 (1.36-3.70) | 0.002 |  |
| Surgical margin status | Neg vs. pos. | 2.91 (1.96-4.33) | 0.000 | 0.626 | 1.52 (0.91-2.54) | 0.111 |  |
| Path. N-stage | pN0 vs. pN1 | 0.53 (0.22-1.31) | 0.169 | 0.523 | - | - | - |

*Meth.: Methylation. Path.: Pathologic. HR: Hazard ratio. CI: Confidence interval.*
