# Supplementary material for: Epigenetic silencing of MEIS2 in prostate cancer recurrence
Source: Clin Epigenetics. 2019 Oct 22;11:147. doi: 10.1186/s13148-019-0742-x (PMC6805635; doi:10.1186/s13148-019-0742-x)
Supplement: Supplementary file 13 — Additional file 13: Table S9. Uni- and multivariate cox regression of cg25381383 in the TCGA 450K cohort (n=389 patients). BCR was used as end-point. Meth: Methylation. Path.: Pathologic. HR: Hazard ration. CI: Confidence interval. [file 13148_2019_742_MOESM13_ESM.docx]

Additional file 13: Table S9

*Uni- and multivariate cox regression of cg25381383 in the TCGA 450K cohort (n=389 patients).*

| Variable | | Univariate | | | Multivariate | | |
| --- | --- | --- | --- | --- | --- | --- | --- |
|  |  | HR (CI) | p-val | C-index | HR (CI) | p-val | C-index |
| Cg25381383 | Low vs. high | 2.62 (1.44-4.77) | 0.002 | 0.643 | 2.09 (1.14-3.82) | 0.017 | 0.717 |
| Gleason score | <7 | 1 | | 0.646 | 1 | |  |
|  | =7 | 2.77 (0.63-12.13) | 0.176 |  | 1.54 (0.34-6.90) | 0.572 |  |
|  | >7 | 7.53 (1.80-31.58) | 0.006 |  | 3.38 (0.77-14.78) | 0.106 |  |
| Path. T-stage | T2 vs. T3 | 6.02 (2.16-16.81) | 0.001 | 0.617 | 3.89 (1.34-11.27) | 0.012 |  |
| Surgical margin status | Neg. vs. pos. | 1.47 (0.82-2.65) | 0.198 | 0.542 | - | - | - |

*BCR was used as end-point. Meth: Methylation. Path.: Pathologic. HR: Hazard ration. CI: Confidence interval.*
